# Supplementary material for: High FREM2 Gene and Protein Expression Are Associated with Favorable Prognosis of IDH-WT Glioblastomas
Source: Cancers (Basel). 2019 Jul 27;11(8):1060. doi: 10.3390/cancers11081060 (PMC6721429; doi:10.3390/cancers11081060)
Supplement: Supplementary file 1 [file cancers-11-01060-s001.pdf]

Supplementary Materials

High *FREM2* Gene and Protein Expression Are Associated with Favorable Prognosis of *IDH*-WT Glioblastomas

Ivana Jovčevska, Alja Zottel, Neja Šamec, Jernej Mlakar, Maxim Sorokin, Daniil Nikitin, Anton A. Buzdin and Radovan Komel

Table S1. Detailed clinical information of the glioma patients. F, female; M, male; KPS, Karnofsky Performance Scale score; OS, overall survival (where there is no number, patients are still alive); WHO, World Health Organization; GTR, gross total resection (or complete macroscopic tumor removal); *IDH*, isocitrate dehydrogenase; *ATRX*, alpha-thalassemia/mental retardation syndrome, X-linked; *TP53*, tumor protein p53; N/A, test not needed or not performed.

Gender	Age	KPS	OS (Months)	Diagnosis	WHO Grade	Anatomic Location	GTR	1p/19q Codeletion	*IDH1* R132H Status	*ATRX*	*TP53*
F	53	70		Oligodendroglioma	II	Parietal lobe, right	-	+	+	No loss	Wild type
M	51	100	21	Diffuse astrocytoma	II	Frontal lobe, right	-	-	-	No loss	Wild type
M	44	90		Diffuse astrocytoma	II	Insular cortex, right	-	-	+	Loss	Wild type
F	50	80		Oligodendroglioma	II	Frontal lobe, left	-	+	+	No loss	Wild type
M	33	60	23	Diffuse astrocytoma	II	Temporal lobe, right	-	N/A	+	Loss	Wild type
M	28	100		Diffuse astrocytoma	II	Frontal lobe, right	-	N/A	+	Loss	Wild type
F	52	80		Diffuse astrocytoma	II	Frontal lobe, right	+	N/A	+	Inconclusive	Mutated
M	25	100		Diffuse astrocytoma	II	Temporal lobe, right	+	-	+	Loss	Wild type
M	34	90	7	Diffuse astrocytoma	II	Frontal lobe, left	-	-	N/A	N/A	N/A
M	35	100		Diffuse astrocytoma	II	Frontal lobe, right	-	19q deletion	+	Loss	Inconclusive
F	48	80		Diffuse astrocytoma	II	Insular cortex, right	-	-	-	Loss	Wild type
M	29	70		Anaplastic astrocytoma	III	Insular cortex, left	-	N/A	+	Loss	Mutated

M	34	90		Anaplastic astrocytoma	III	Frontal lobe, right	-	N/A	+	Loss	Mutated
M	48	80	19	Glioblastoma	IV	Temporal lobe, right	-	-	-	No loss	Wild type
F	81	40	2	Glioblastoma	IV	Frontal lobe, left	+	N/A	-	No loss	Wild type
M	68	80		Glioblastoma	IV	Frontal lobe, right	-	N/A	-	No loss	Mutated
M	45	100	18	Glioblastoma	IV	Temporal lobe, left	-	N/A	-	No loss	Wild type
F	72	60	5	Glioblastoma	IV	Frontal lobe, left	-	N/A	-	No loss	Wild type
F	58	70		Giant cell glioblastoma	IV	Temporal lobe, left	-	N/A	-	N/A	Mutated
M	75	70	10	Glioblastoma	IV	Occipital lobe, right	+	N/A	-	N/A	Mutated
M	64	100	17	Glioblastoma	IV	Parietal-occipital lobe, right	+	N/A	-	No loss	Wild type
M	59	40		Glioblastoma	IV	Parietal lobe, right	-	N/A	-	No loss	Wild type
F	72	60	1	Glioblastoma	IV	Frontal lobe, left	+	N/A	-	No loss	Wild type
M	52	50	15	Glioblastoma	IV	Temporal lobe, left	-	N/A	-	Loss	Wild type
M	41	100	24	Gliosarcoma	IV	Temporal lobe, right	-	N/A	-	No loss	Mutated

© 2019 by the authors. Licensee MDPI, Basel, Switzerland. This article is an open access article distributed under the terms and conditions of the Creative Commons Attribution (CC BY) license (http://creativecommons.org/licenses/by/4.0/).
